# 746. A Bacteriophage-Based Validation of a Personal Protective Equipment Doffing Procedure to be Used with High Consequence Pathogens

**DOI:** 10.1093/ofid/ofad500.807

**Published:** 2023-11-27

**Authors:** Kylie Burke, Brandon A Berryhill, Colleen S Kraft, Andrew Smith, Jill Morgan, Jessica Tarabay, Josia Mamora, Jay Varkey, Joel Mumma, Darryl Grant, Lindsay M Busch, Jessica Carag

**Affiliations:** Emory University School of Medicine, Decatur, Georgia; Emory University, Atlanta, Georgia; Emory University, Atlanta, Georgia; Emory University, Atlanta, Georgia; Emory University Hospital, Atlanta, Georgia; Emory Healthcare, Atlanta, Georgia; Emory University Hospital, Atlanta, Georgia; Emory University School of Medicine, Decatur, Georgia; Emory University School of Medicine, Decatur, Georgia; Emory University School of Medicine, Decatur, Georgia; Emory University School of Medicine, Decatur, Georgia; Emory University School of Medicine, Decatur, Georgia

## Abstract

**Background:**

Personal protective equipment (PPE) is the cornerstone of healthcare worker (HCW) safety and prevention of the spread of pathogens. To limit the potential risk to HCWs, healthcare institutions have employed infection control policies and procedures. When treating patients with high consequence infectious diseases, such as Ebola virus disease, HCWs wear complex personal protective equipment (PPE) ensembles that cover their entire body with multiple layers. These layers build redundancy to ensure protection of the HCWs' skin and clothing. Manufacturers typically provide recommendations for donning and doffing individual PPE pieces, but protocols differ between healthcare institutions for PPE ensembles. These PPE ensembles and their associated doffing protocols are rarely tested empirically.

**Methods:**

We reviewed footage of the doffing protocol currently employed at a Regional Emerging Special Pathogen Treatment Center. From this analysis we identified potential contamination pathways which we verified by using a fluorescent pathogen proxy. To account for viable pathogen susceptibility to alcohol-based disinfectant, HCWs donned the PPE ensemble and were then contaminated with three genetically marked bacteriophages (phage). After completing the doffing protocol, the HCW’s skin and PPE were swabbed to test for the transfer of viable phage to the HCW.

**Results:**

All four of the HCWs that completed the initial doffing protocol had a high recovery of phage from at least one sampling location. Subsequently, we implemented interventions to both the equipment and the doffing procedure. In nine trials with this modified protocol, no viable phage was recovered from any of the HCWs.

**Figure:**
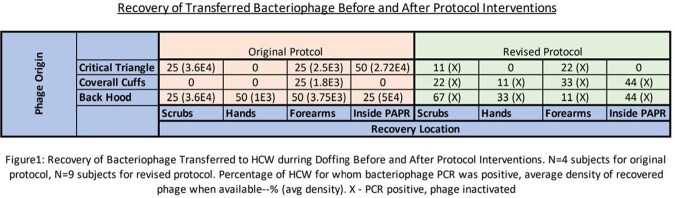

Recovery of Bacteriophage Transferred to HCW during Doffing Before and After Protocol Interventions. N=4 subjects for original protocol, N=9 subjects for revised protocol. Percentage of HCW for whom bacteriophage PCR was positive, average density of recovered phage when available--% (avg density). X- PCR positive, phage inactivated

**Conclusion:**

Our experiments revealed potential contamination pathways that allowed for high densities of viable phage to move from areas of the outermost layers of PPE to the skin and clothing of HCWs. While the individual PPE functioned correctly, the original ensemble and doffing procedure were not effective. Our updated protocol eliminated phage contamination. This study highlights the need for infection prevention protocols to be evaluated in an empirical manner in a framework such as this.

**Disclosures:**

**All Authors**: No reported disclosures

